# A case of COVID-19 with papulovesicular rash that progressed to retiform purpura, accompanied by cherry angiomas

**DOI:** 10.1590/1516-3180.2020.0620.R1.0212020

**Published:** 2021-02-08

**Authors:** Senay Agirgol, Ceyda Çaytemel, Ahmet Şah Kolan, Hüseyin Vural

**Affiliations:** I MD, PhD. Dermatologist, Polyclinic for Dermatology and Venerology, Dermatology Clinic, Haseki Eğitim ve Araştırma Hastanesi, Istanbul, Turkey.; II MD, PhD. Dermatologist, Polyclinic for Dermatology and Venerology, Dermatology Clinic, Haseki Eğitim ve Araştırma Hastanesi, Istanbul, Turkey.; III MD, PhD. Ophthalmologist, Kolan International Hospital, Istanbul, Turkey.; IV MD, PhD. Otolaryngologist, Kolan International Hospital, Istanbul, Turkey.

**Keywords:** COVID-19 [supplementary concept], Purpura, Eczema, Cutaneous, Retiform purpura, Cherry angiomas, Eruptive.

## Abstract

**CONTEXT:**

Various skin manifestations have been reported in coronavirus disease. It may be difficult to determine the etiology of these lesions in view of the increased frequency of handwashing during the pandemic, along with occurrences of irritant contact dermatitis and allergic contact dermatitis due to disinfectant use; usage of herbal medicine and supplements to strengthen the immune system; and urticarial or maculopapular drug eruptions due to COVID-19 treatment. The variety of associated skin manifestations seen with COVID-19 makes it challenging to identify virus-specific skin manifestations. Petechiae, purpura, acrocyanosis and necrotic and non-necrotic purpura, which can be considered as manifestations of vascular involvement on the skin, have been reported.

**CASE REPORT:**

Here, we report a case of eruptive cherry angiomas, which was thought to have developed due to COVID-19, with a papulovesicular rash on distal extremities that progressed over time to reticular purpura.

**CONCLUSION:**

The case presented had a papulovesicular rash at the onset, which evolved to retiform purpura, and eruptive cherry angiomas were observed. It should be kept in mind that dermatological signs may vary in patients with COVID-19.

## INTRODUCTION

Coronavirus disease-2019 (COVID-19) has seriously affected global health since December 2019.^1^ It was declared a pandemic by the World Health Organization (WHO) on April 11, 2020.^2^

The most serious manifestation of the disease is severe acute respiratory failure; however, skin manifestations have been reported with increasing frequency in the literature. Although the pathophysiology of COVID-19 is not yet known, some theories have been suggested. One possibility for its pathogenesis is microvascular injury, for which vascular occlusive manifestations have been detected in biopsies and autopsies.^3^^,^^4^^,^^5^ Manifestations of cutaneous vascular involvement such as petechiae, purpura, acrocyanosis and necrotic and non-necrotic purpura have been reported in association with COVID-19.^5^^,^^6^^,^^7^

Because of the increased frequency of handwashing, along with occurrences of irritant contact dermatitis and allergic contact dermatitis due to disinfectant use, consumption of herbal medicine and supplements to strengthen the immune system prior to infection and the possibility of urticaria or maculopapular drug eruptions post-infection, it may be challenging to determine the etiology of skin lesions. It is difficult to clarify whether these are caused by viruses or are induced by the immune system, and also whether they are virus-specific or nonspecific.^6^^,^^7^ Clinical manifestations can be better understood if there are reports of specific skin lesions that develop through COVID-19.

Here, a case of eruptive cherry angioma accompanied by papulovesicular rash on the distal extremities that evolved to reticular purpura, which was thought to have developed secondary to COVID-19, is presented.

## OBJECTIVE

Petechiae and purpuras are generally thought to be a symptom of serious systemic disease and these lesions have been reported in relation to coronavirus infection. Information about COVID-19-related skin symptoms is expanding day by day. Petechiae relating to COVID-19, which are generally observed in severe cases, can also be seen in uncomplicated cases, as in our patient. We believe that the unique clinical course of our patient will provide guidance if a similar situation were to develop.

## CASE REPORT

A 52-year-old male patient came to our clinic for a consultation regarding rashes on his fingers, arms and legs. It was learned from the patient’s story that he had been screened for the novel coronavirus (2019-nCoV) because his wife had tested positive two days previously. A reverse transcription polymerase chain reaction (RT-PCR) test for severe acute respiratory syndrome coronavirus 2 (SARS-CoV-2) was found to be positive. The samples were obtained using a nasopharyngeal swab.

**Figure 1.**
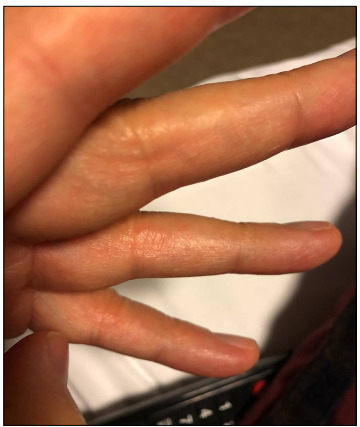
Vesicles on the sides of the fingers.

He had complaints of mild weakness and myalgia and he had been using tablets of diltiazem (120 mg/day), doxazosin (4 mg/day), atorvastatin (10 mg/day) and acetylsalicylic acid (100 mg/day) for five years due to essential hypertension and coronary artery disease. The patient did not complain of coughing or dyspnea, and no lung involvement was detected on computed tomography (CT). In laboratory tests, acute-phase reactants were found to be within the normal range: procalcitonin 0.005 ng/ml, sedimentation 12 mm/h and C-reactive protein (CRP) 8 mg/l. The patient was accepted as a COVID-19 case and treatments with hydroxychloroquine (600 mg BID for one day and then 400 mg BID for the following five days) and azithromycin (500 mg daily for five days) were started. Two days after the onset of constitutional symptoms, vesicular skin lesions started to develop on the finger’s side as can be seen in Figure 1.

Informed consent was obtained from the patient for publication of clinical pictures, and permission to report on this case was granted by the local ethics committee.

**Figure 2.**
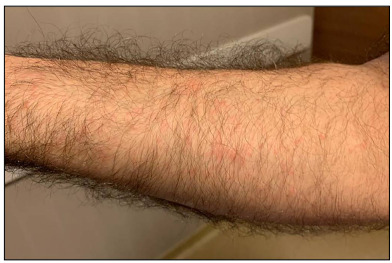
Millimetric erythematous papulovesicles, particularly on the flexor side of the arms.

**Figure 3.**
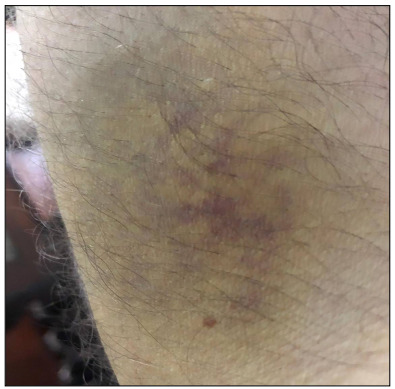
Retiform petechial-purpuric appearance beginning to occur on the patient’s arm.

**Figure 4.**
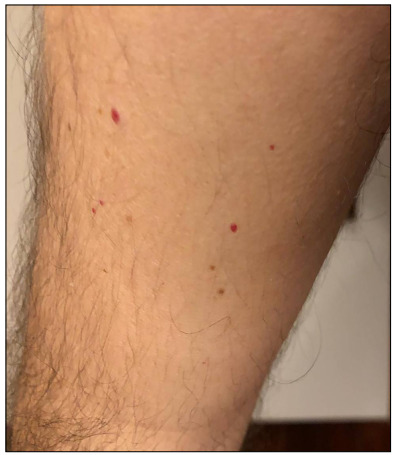
Cherry angioma-like lesions on the medial aspect of the upper arm, extending to the armpit.

The patient’s first dermatological signs consisted of vesicles on the sides of his fingers (Figure 1) and millimetric erythematous papulovesicular eruptions, which were concentrated on the extensor aspect of the lower legs and flexor aspect of the arms (Figure 2). Initially, the patient was evaluated as having irritant contact dermatitis, and administration of mometasone furoate ointment and cetirizine tablet (20 mg/day) was started. After three days, it was noticed that the patient’s existing lesions began to acquire a petechial-purpuric appearance (Figure 3) and cherry angioma-like lesions appeared on his upper arms, extending from the inner side to the armpit, and on the upper lateral aspects of the trunk (Figure 4).

In laboratory tests, antinuclear antibody levels, antithrombin-3 levels, complete blood count, liver function tests and coagulation parameters were found to be within the normal range and conditions that cause vasculitis were ruled out. No histopathological examination of the skin lesions was performed because the patient did not agree to undergo a biopsy.

The patient developed severe weakness and severe muscle pain in his legs, which could be interpreted as restless leg syndrome. The skin manifestations gradually regressed leaving a reticular purpuric appearance. Some of the cherry angioma-like lesions disappeared after a brown crust had formed, while others persisted.

## DISCUSSION

The variety of skin manifestations associated with COVID-19 makes it difficult to identify virus-specific skin manifestations. In the literature, several classifications have been made using skin manifestations associated with COVID-19.^5^ The cutaneous manifestations of the disease have been grouped into five clinical patterns: 1) pseudo-chilblain; 2) vesicular; 3) urticarial; 4) maculopapular; and 5) livedo/necrosis. These patterns are now being confirmed by other authors.^5^^,^^6^^,^^7^^,^^8^

Attempts to understand the clinical-anatomopathological patterns of COVID-19 are being made. In a study examining skin and lung tissue from five patients, the thrombotic microvascular injury was detected through biopsies. It was claimed that the presence of livedo or necrosis correlated with greater severity of COVID-19.^5^ In addition, vascular lesions have been reported in younger and milder cases.^6^

In the literature, histopathological examination of a biopsy taken from a case with retiform purpura showed thrombi in small cutaneous vessels. There was also C3 and C9 deposition, thereby demonstrating complement activation.^5^ In the present case, no biopsy was taken because the patient refused to undergo this procedure. However, other systemic causes of purpura, such as thrombocytopenia and bleeding diathesis, were investigated and ruled out. Severe pain was present in the patient’s leg muscles, which may suggest that formation of vasculopathy is not limited only to cutaneous vessels but also affects larger vessels. In the present case, no thrombus or pneumonia was detected in the lungs, which confirmed the previously reported observation that purpura might also be seen in mild cases.^7^

Cherry angiomas are generally seen on the trunk in middle-aged adults. Their pathogenesis is not well understood. It has been suggested that immune dysregulation of the skin could be a predisposing factor for the development of eruptive cherry angiomas.^9^ Cherry angiomas in COVID-19 cases have only been rarely reported in the literature. Eruptive cherry angioma of the back has been reported.^7^ Cherry angiomas were noticed in the case of our patient when the lesions gained reticular purpuric characteristics. The simultaneous occurrence of two types of lesions may indicate a common etiopathogenesis. In a previous study, it was determined histopathologically that angiotensin-converting enzyme 2 (ACE2) receptors were secreted from endothelial cells and that the virus caused endotheliitis through this receptor.^10^ Retiform purpura and eruptive cherry angioma formation in the patient could be caused by endotheliitis. The occurrences of lesions on the patient’s extremities made it easier to recognize and monitor them.

Endothelial cell involvement in COVID-19 patients was recently observed. It was demonstrated histologically that these cells express ACE2 receptors, through which SARS-CoV-2 manages to infect the host, accompanied by viral elements and inﬂammatory cells. These ﬁndings suggest that SARS-CoV-2 infection may produce endotheliitis in different organs, including the skin, as a direct effect from the presence of the virus and host’s inﬂammatory response.^10^ Maculopapular rash, one of the skin manifestations associated with coronavirus, is the most frequently reported skin manifestation, although cases of retiform purpura and, rarely, cherry angioma have also been reported (Table 1).

**Table 1.** A systematic review of the literature.

Database	Search strategy	Results
MEDLINE/PubMed	(Skin manifestations) AND Purpura (retiform) AND (COVID-19)	4 reviews 4 original articles 3 case reports
	Cherry angiomas	1 original article
EMBASE	Skin manifestations) AND Purpura (retiform) AND (COVID-19)	4 reviews 4 original articles 3 case reports
	Cherry angiomas	1 original article
LILACS	Skin manifestations) AND Purpura (retiform) AND (COVID-19)	4 reviews 4 original articles 3 case reports
	Cherry angiomas	1 original article

The search in these databases was conducted in October 2020.

## CONCLUSION

Our case presented with a papulovesicular lesion at the onset, which progressed to retiform purpura, accompanied by eruptive cherry angiomas that became partly crusted and regressed, according to the patient. We did not find any cases with a similar course in the literature available. It should be kept in mind that dermatological signs may vary in patients with COVID-19. Clinicians should therefore be careful about this.
